# Transcription factor FfMYB15 regulates the expression of cellulase gene *FfCEL6B* during mycelial growth of *Flammulina filiformis*

**DOI:** 10.1186/s12934-022-01932-z

**Published:** 2022-10-17

**Authors:** Zongqi Liu, Bing Deng, Hui Yuan, Benfeng Zhang, Jingyu Liu, Junlong Meng, Mingchang Chang

**Affiliations:** 1 College of Food Science and Engineering, Shanxi Agricultural University, 1 Mingxian South Road, Taigu, 030801 Shanxi Province China; 2 Collaborative Innovation Center of Advancing Quality and Efficiency of Loess Plateau Edible Fungi, Taigu, 030801 Shanxi China; 3 Shanxi Key Laboratory of Edible Fungi for Loess Plateau, Shanxi Agricultural University, Taigu, 030801 Shanxi China

**Keywords:** *Flammulina filiformis*, MYB, Transcriptional regulation, Cellulase, Cellulose degradation

## Abstract

**Background:**

Cellulose degradation can determine mycelial growth rate and affect yield during the growth of *Flammulina filiformis*. The degradation of cellulose requires the joint action of a variety of cellulases, and some cellulase-related genes have been detected in mushrooms. However, little is known about the transcriptional regulatory mechanisms of cellulose degradation.

**Results:**

In this study, FfMYB15 that may regulate the expression of cellulase gene *FfCEL6B* in *F. filiformis* was identified. RNA interference (RNAi) showed that *FfCEL6B* positively regulated mycelial growth. Gene expression analyses indicated that the expression patterns of *FfCEL6B* and *FfMYB15* in mycelia cultured on the 0.9% cellulose medium for different times were similar with a correlation coefficient of 0.953. Subcellular localization and transcriptional activity analyses implied that FfMYB15 was located in the nucleus and was a transcriptional activator. Electrophoretic mobility shift assay (EMSA) and dual-luciferase assays demonstrated that FfMYB15 could bind and activate *FfCEL6B* promoter by recognizing MYB cis-acting element.

**Conclusions:**

This study indicated that *FfCEL6B* played an active role in mycelial growth of *F. filiformis* and was regulated by FfMYB15.

**Supplementary Information:**

The online version contains supplementary material available at 10.1186/s12934-022-01932-z.

## Background

The degradation and utilization of cellulose by fungi not only plays a positive role in the carbon cycle, but also brings great commercial value in industrial production [1–3]. At present, many scholars have explored filamentous fungi, such as *Trichoderma reesei, Penicillium oxalicum*, and *Aspergillus niger* in enzyme production [4–6]. By using cellulose as raw materials to efficiently produce cellulase, it was found that the mycelial growth in the process of decomposing cellulose was promoted [4–6]. As macro-fungi, mushrooms are also an important agent of degradation of cellulose [7]. During their growth, mushrooms can degrade cellulose in cultivation substrate by secreting a variety of extracellular enzymes, thus providing energy for their growth and development [8]. In production, materials with a high cellulose content are often used as cultivation substrate to increase the yield of mushrooms [9].

The degradation mechanism of cellulose was found to be a complex process which is jointly regulated by a variety of cellulases [10]. Currently, cellulase genes encoding endoglucanase (EGL), cellobiohydrolase (CBH), and β-glucosidase (BGL) have been detected in many filamentous fungi [11–13]. Many transcription factors (TFs), such as ACE3 (*Trichoderma reesei*), ATF1 (Ascomycetes), ACE2 (*T. reesei*), CLR-4 (Ascomycete fungi), etc., were found to regulate cellulase genes and participate in cellulose degradation [14–17]. However, only a few genes that encode cellulases, such as *LeCEL7A* (*Lentinus edodes*), *LeCEL6B* (*L. edodes*), *PaCEL3A* (*Polyporus arcularius*), and *AbCEL3* (*Agaricus bisporus*), have been detected in mushrooms [18–20].

CBH is considered a key component of the cellulase system, and its main product acting on cellulose is cellobiose [21]. The *CEL6B* gene encoding CBH has been identified in *L. edodes* [22, 23]. Previous study found that *LeCEL6B* was involved in the degradation of cellulose and promotes mycelial growth in *L. edodes* [23], however, the transcriptional regulation mechanism of TFs that can regulate cellulose degradation in mushrooms remains unclear.

The myeloblastosis (MYB) transcription factor family, characterized by a highly conserved MYB DNA-binding domain, is widely distributed in eukaryotic organisms [24]. MYB TFs have been confirmed to be participated in biological processes such as secondary metabolism, cell differentiation, and in the growth and development of plants and fungi [25–27]. In addition, they were found to be participated in the biosynthetic pathway of cellulase [28, 29]. In previous studies, the transcription factor MYB72 was involved in the regulation of β-glucosidase BGLU42 in *Pseudomonas simiae* in *Arabidopsis thaliana* [28]. OsMPS (a MYB TF) was involved in regulating the expression of endoglucanase genes *OsGLU14* and *OsGLU5* in rice [29]. The MYB motif in the promoter of cellulase *CelB* in *Dictyostelium discoideum* might bind to MYB protein to regulate the *CelB* gene expression [30]. MYB TFs have been identified in mushrooms (*Ganoderma lucidum* and *Pleurotus ostreatus*) and show the potential to participate in mushroom growth and development by interacting with specific genes [27, 31]. They have become a focus of much research into mushroom growth and development, however, the involvement of them in the expression of cellulase genes related to cellulose degradation in mushroom growth, especially in industrially cultivated mushrooms, has not yet been clarified.

*Flammulina filiformis* is not only a widely cultivated mushroom in East Asia, but also one of the five largest industrially cultivated mushrooms in the world [32]. It can be used as an ideal research material, due to its short growth cycle and simple cultivation method, and the availability of transcriptomic data and genome at different developmental stages [33, 34]. A previous study has found that *CEL6B* played a role in promoting the mycelial growth of *L. edodes* [23]. Whether it plays the same role in *F. filiformis* has not been studied. The sequence information of *FfCEL6B* was obtained by keyword search in the annotated genome database of *F. filiformis* and sequence alignment with *CEL6B* in *L. edodes* (GenBank: XP-046091961.1). RNAi showed that *FfCEL6B* was actively involved in the mycelial growth of *F. filiformis*. However, the transcriptional regulation of *FfCEL6B* is unclear.

Our previous study showed that a total of 13 MYB TFs were identified in *F. filiformis*, which were involved in mycelial growth and fruiting body development. Among them, the gene expression levels of the three MYB TFs in the mycelium stage were higher than that in other growth and development stages [35]. This study found that the expression patterns of *FfCEL6B* and *FfMYB15* in mycelia cultured on the 0.9% cellulose medium for different times were similar. The possible association of FfMYB15 regulating the expression of *FfCEL6B* was further explored. Our study provided a new basis for revealing the transcriptional regulation mechanism of cellulose degradation during the growth of *F. filiformis*.

## Results

### Changes in cellulase activity during mycelial growth of *F. filiformis*

**Fig. 1:**
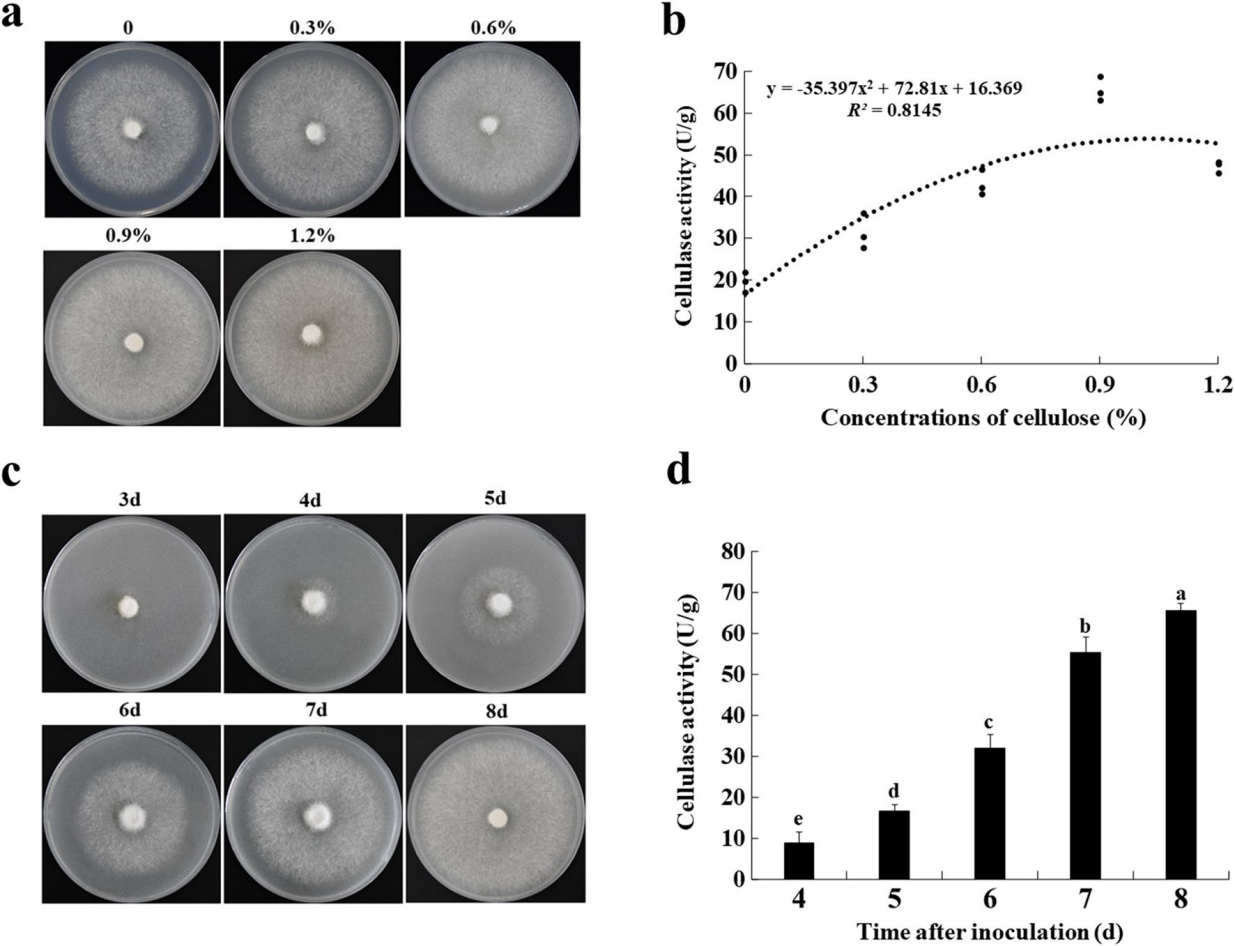
Cellulase promotes the mycelial growth of *F. filiformis*. **a** Changes in mycelial growth rate on the 0, 0.3%, 0.6%, 0.9%, and 1.2% cellulose medium for 8 d. **b** Cellulase activity of mycelia cultured on the medium with 0, 0.3%, 0.6%, 0.9%, and 1.2% cellulose for 8 days. **c** Mycelial growth in the 0.9% cellulose medium for 3, 4, 5, 6, 7, and 8 d. **d** Cellulase activity of mycelia cultured on the 0.9% cellulose medium for 4, 5, 6, 7, and 8 d. Each value represents the mean ± SD (n = 3), and different lowercase letters denote statistical significance (*P* < 0.05)

To investigate the effect of cellulase activity on the mycelial growth of *F. filiformis*, *F. filiformis* mycelia were cultured on a culture medium added with different concentrations of cellulose. Glucose was not added to the medium to make the mycelium secrete cellulase. Under the same culture conditions, *F. filiformis* mycelia were cultured for eight days. It was found that the mycelial growth rate of *F. filiformis* on the culture medium containing cellulose was higher than that without cellulose. With the increase of cellulose content, the mycelial growth rate gradually accelerated. When the cellulose content reached 0.9%, the mycelial growth rate was the highest, but the mycelial growth rate decreased with the increase in the amount of cellulose (Fig. 1a).

The cellulase activities of mycelia collected on the medium with 0, 0.3%, 0.6%, 0.9%, and 1.2% cellulose for 3, 4, 5, 6, 7, and 8 d were determined by using a 3,5-dinitrosalicylic acid method [36]. It was found that the cellulase activity secreted by mycelia increased with the increase in the mycelial growth rate. When mycelia were cultured on the 0.9% cellulose medium, the growth rate was the fastest, and the cellulase activity was the highest at 65.5 u/g. With the continuous addition of cellulose, the cellulase activity decreased (Fig. 1b). The correlation coefficient between mycelial growth and cellulase activity was 0.966 by the Pearson correlation coefficient method [37], which indicated that cellulase activity was positively correlated with the mycelial growth rate.

In addition, cellulase activities showed a gradually increasing trend during the growth of mycelia in the medium with different concentrations of cellulose (Additional file 1: Fig. S1). Among the experimental groups, the mycelial cellulase activity in the 0.9% cellulose medium was the most representative. The changes in the mycelium and the cellulase activities during the mycelial growth are shown in Figs. 1c and d, respectively. These results indicated that cellulase can participate and promote mycelial growth of *F. filiformis*.

### Cloning and sequence analysis of *FfCEL6B*

**Fig. 2:**
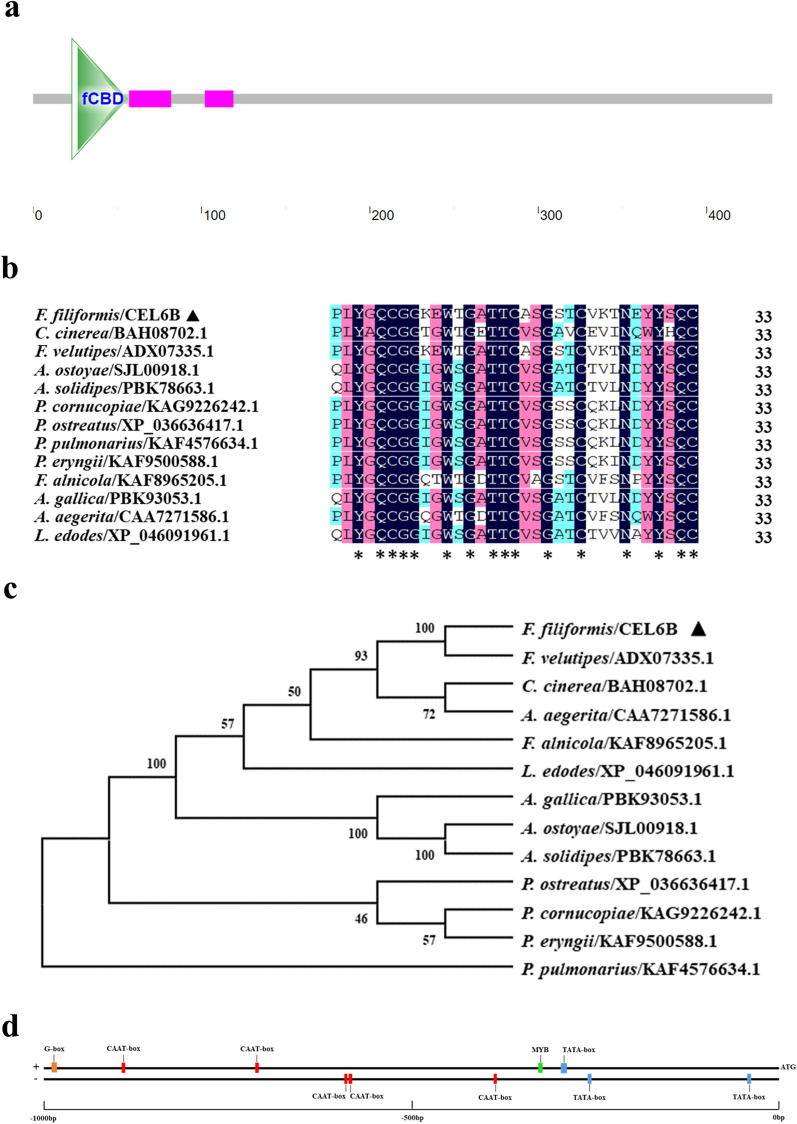
Bioinformatic analysis of *FfCEL6B*. **a** Schematic diagram of fCBD domain of FfCEL6B. **b** fCBD protein sequence alignment. fCBD homologous proteins are from *Pleurotus cornucopiae*, *Coprinopsis cinerea*, *Pleurotus eryngii*, *Flammulina velutipes*, *Armillaria ostoyae*, *Armillaria solidipes*, *P. ostreatus*, *Flammula alnicola*, *Pleurotus pulmonarius*, *Armillaria gallica*, *Agrocybe aegerita*, and *Lentinula edodes*. Asterisks represent conserved amino acids. **c** Phylogenetic relationships between FfCEL6B and sequence alignment species. The triangle denotes FfCEL6B in *F. filiformis*. **d** Schematic representation of major cis-acting elements in the *FfCEL6B* promoter. “ + ” denotes the sense sequence and “−” represents the antisense strand

To obtain the sequence information of *FfCEL6B* gene, it was cloned and bioinformatics analysis was performed, wherein the bioinformatic analysis included molecular weight, theoretical isoelectric point (pI), DNA domain prediction, sequence alignment, phylogenetic analysis, and promoter analysis. According to the nucleotide sequence, specific primers were designed and *FfCEL6B* was cloned (Additional file 1: Fig. S2). The total length of the open reading frame of *FfCEL6B* was 1320 bp encoding a polypeptide of 440 amino acids. The molecular weight and pI of *FfCEL6B* were 90.67 kDa and 4.90. The fungal-type cellulose-binding domain (fCBD) was annotated at amino acid residues 23–56 (Fig. 2a). fCBD combined with cellulose has the function of decomposing cellulose [38].

FfCEL6B homologs can be found in *Coprinopsis cinerea* (model mushroom species), *P. ostreatus*, *Pleurotus eryngii*, *Armillaria gallica*, *Agrocybe aegerita* (industrially cultivated mushrooms), and other mushroom species. Their sequences in the fCBD were conserved (Fig. 2b). The phylogenetic tree showed that FfCEL6B was most closely related to *F. velutipes* (Fig. 2c).

TATA box, CAAT box, and G-box were scanned in the sequence analysis of the *FfCEL6B* promoter. The TATA box was the core promoter element around -30 from the start of transcription, proving its existence on many eukaryotic gene-promoter regions [39]. The CAAT box was mostly present in promoter and enhancer regions. G-box participated in light responsiveness [40]. In addition, the MYB motif was also found, which was predicted to be at the 671 bp position of the sense sequence (+) and the sequence was CAACCA (Fig. 2d). This finding suggested that MYB TFs may be involved in the regulation of *FfCEL6B*.

### *FfCEL6B* participates in the mycelial growth of *F. filiformis*

**Fig. 3:**
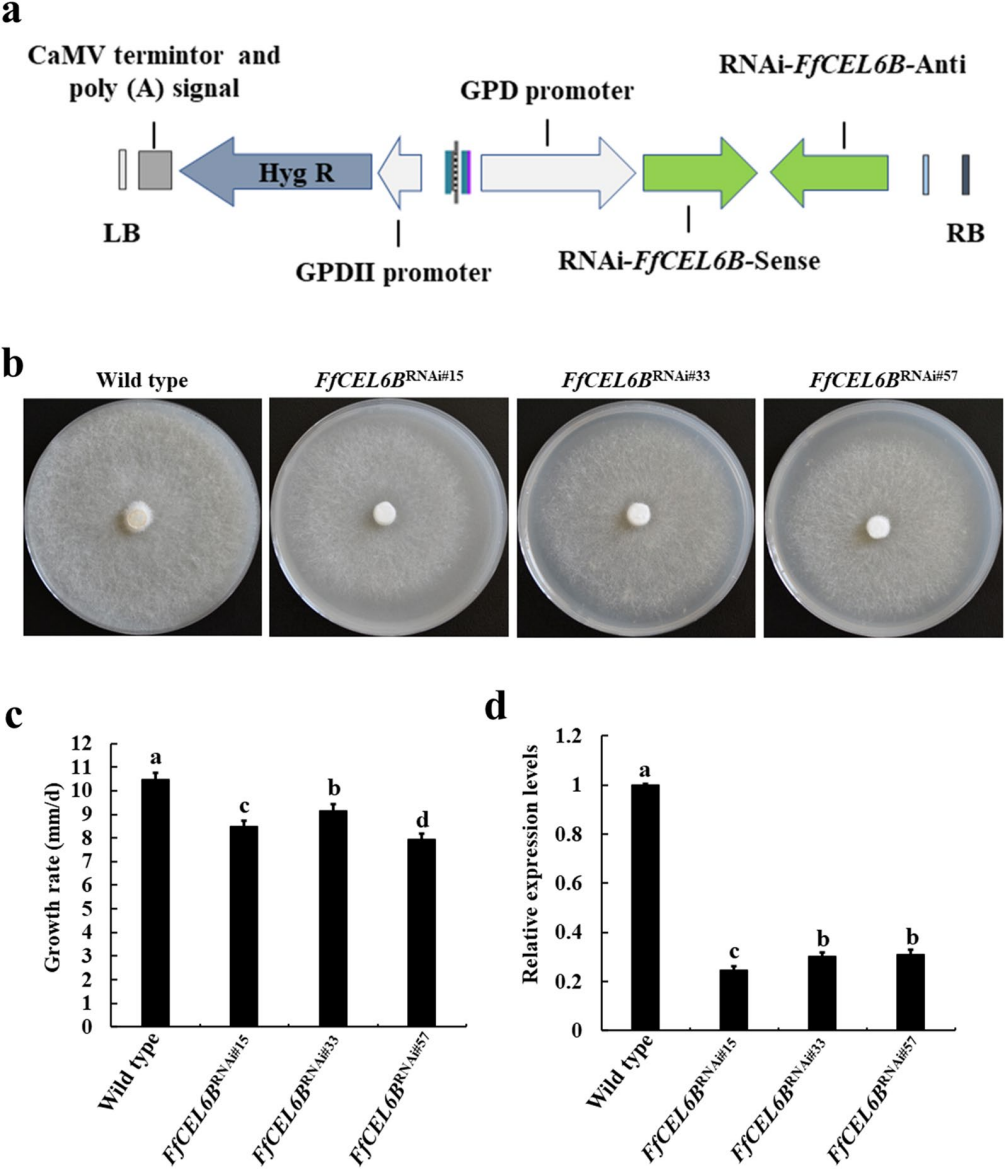
Effects of *FfCEL6B* knockdown on mycelial growth on the 0.9% cellulose medium. **a** The structural diagram of RNAi‑*FfCEL6B* plasmid. **b** Colony morphology of wild type, *FfCEL6B*^RNAi#15^, *FfCEL6B*^RNAi#33^, and *FfCEL6B*^RNAi#57^ strains cultured on the 0.9% cellulose medium for 8 d. **c** Growth rate of *FfCEL6B* transformants. Each value represents the mean ± SD (n = 3). ANOVA and Duncan’s multiple range tests (*P* < 0.05). **d** Expression levels of *FfCEL6B* were detected in *FfCEL6B*^RNAi#15^, *FfCEL6B*^RNAi#33^, and *FfCEL6B*.^RNAi#57^ strains. The expression level of *FfCEL6B* in wild type was set to 1. Each value represents the mean ± SD (n = 3). ANOVA and Duncan’s multiple range tests (*P* < 0.05)

To study the effect of *FfCEL6B* on the mycelial growth rate of *F. filiformis*, RNA interference tests were conducted on *FfCEL6B*. The structural diagram of RNAi‑*FfCEL6B* plasmid is shown in Fig. 3a. RNAi of *FfCEL6B* indicated that the growth rates of RNAi strains were lower than that of the wild-type strain under the same culture conditions (Figs. 3b, c). Expression levels of *FfCEL6B* were detected in three RNAi transformants cultured in the 0.9% cellulose medium. Compared to the wild type, the transcription levels of *FfCEL6B* in *FfCEL6B*^RNAi#15^, *FfCEL6B*^RNAi#33^, and *FfCEL6B*^RNAi#57^ transformants were decreased by 75.3%, 69.7%, and 69%, respectively (Fig. 3d). These results suggested that *FfCEL6B* positively regulated the mycelial growth in *F. filiformis*.

### Cloning and sequence analysis of *FfMYB15*

**Fig. 4:**
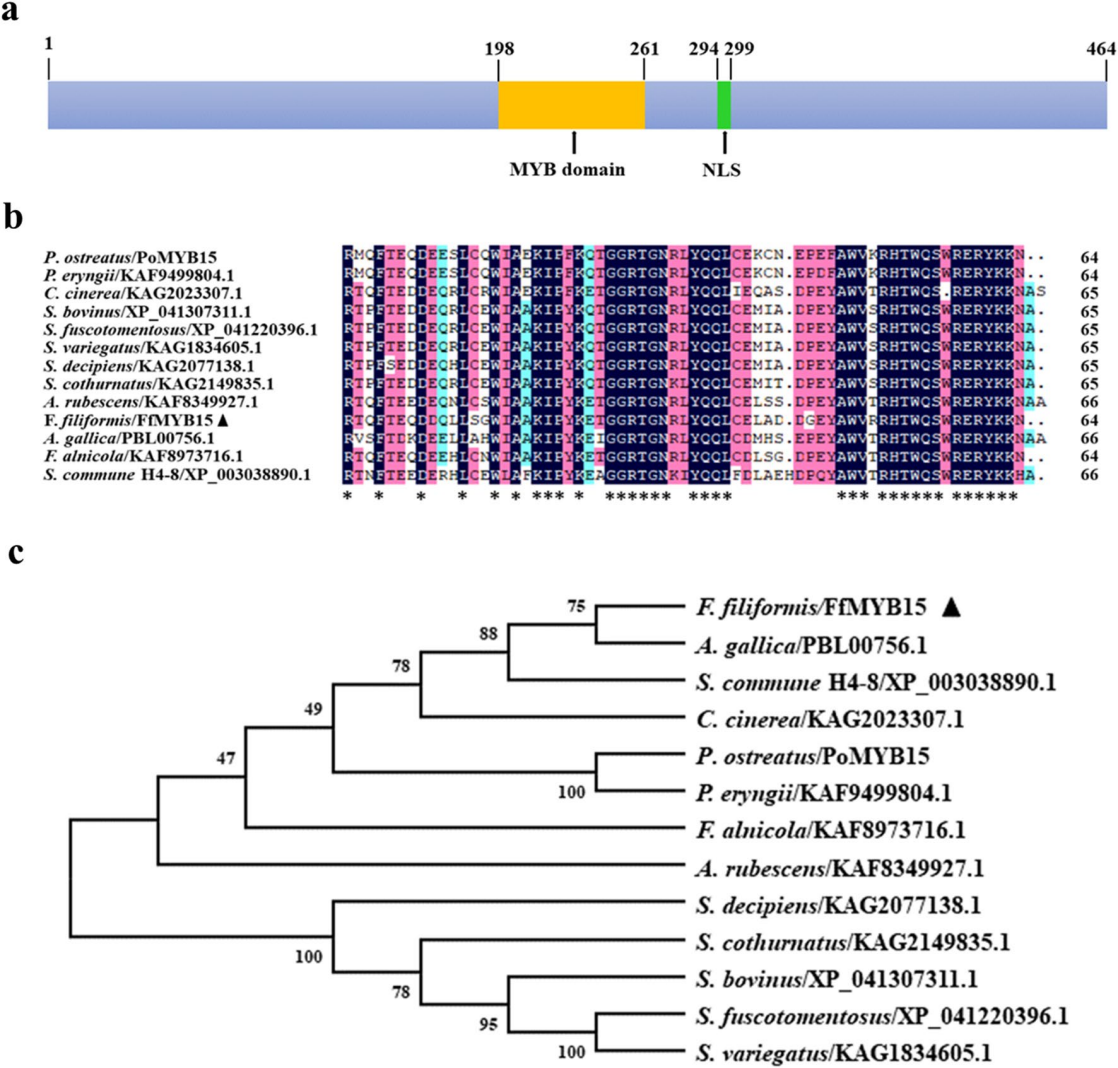
Bioinformatic analysis of *FfMYB15*. **a** Schematic diagram of MYB domain and NLS of FfMYB15. **b** MYB protein sequence alignment. MYB homologous proteins are from *P. ostreatus*, *Pleurotus eryngii*, *Coprinopsis cinerea*, *Suillus bovinus*, *Suillus fuscotomentosus*, *Suillus variegatus*, *Suillus decipiens*, *Suillus cothurnatus*, *Amanita rubescens*, *Armillaria gallica*, *Flammula alnicola*, and *Schizophyllum commune* H4–8. Asterisks represent conserved amino acids. **c** Phylogenetic relationships between FfMYB15 and sequence alignment species. The triangle represents FfMYB15 *in F. filiformis*

To obtain the sequence information of *FfMYB15* gene, it was cloned and bioinformatic analysis was performed, wherein the bioinformatic analysis included molecular weight, pI, nuclear localization sequence (NLS), DNA domain prediction, sequence alignment, and phylogenetic analysis. According to the nucleotide sequence, specific primers were designed and *FfMYB15* was cloned (Additional file 1: Fig. S3). The total length of the open reading frame of *FfMYB15* was 1392 bp encoding a polypeptide of 464 amino acids. The molecular weight and pI of *FfMYB15* were 96.68 kDa and 7.66. NLS was annotated at amino acid residues 294–299, which could interact with the nuclear carrier so that the protein was transported into the nucleus. The MYB domain was annotated at amino acid residues 198–261 (Fig. 4a).

FfMYB15 homologs could be found in *C. cinerea*, *Schizophyllum commune* (model mushroom species), *P. ostreatus*, *P. eryngii*, *A. gallica* (industrially cultivated mushrooms), and other mushroom species. Their sequences in the MYB domain were conserved (Fig. 4b). The phylogenetic tree showed that FfMYB15 was most closely related to *A. gallica* (Fig. 4c).

### *FfCEL6B* and *FfMYB15* gene expression analyses

**Fig. 5:**
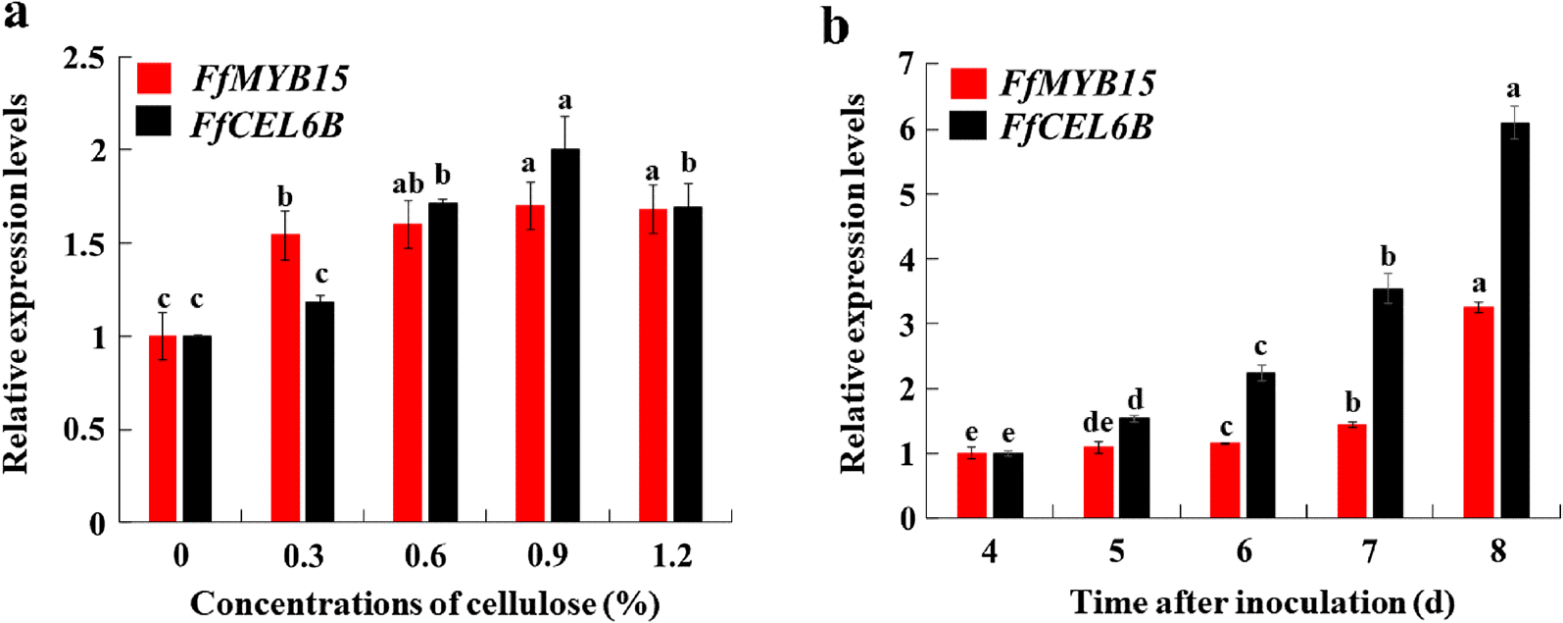
*FfCEL6B* and *FfMYB15* gene expression analyses. **a** *FfCEL6B* and *FfMYB15* expression levels in mycelia cultured on the medium with 0, 0.3%, 0.6%, 0.9%, and 1.2% cellulose for 8 days. *FfCEL6B* and *FfMYB15* expression levels on the medium with no cellulose added were set to 1. **b** The expression levels of *FfCEL6B* and *FfMYB15* in mycelia cultured on the 0.9% cellulose medium for 4, 5, 6, 7, and 8 days. The expression levels of *FfCEL6B* and *FfMYB15* at 4 d were set to 1. Each value represents the mean ± SD (n = 3). ANOVA and Duncan’s multiple range tests (*P* < 0.05)

To understand the relationship between different expression patterns of *FfCEL6B* and *FfMYB15*, the levels of expression of *FfCEL6B* and *FfMYB15* in mycelia cultured on the medium with 0, 0.3%, 0.6%, 0.9%, and 1.2% cellulose for 8 days were detected. It was found that the levels of expression of the *FfCEL6B* and *FfMYB15* in mycelium on the cellulose medium were higher than those on the medium without cellulose. When adding 0.9% cellulose, levels of expression of *FfCEL6B* and *FfMYB15* were the highest, which were 2.00 and 1.70 times the control mycelium level of expression, respectively. When the content of added cellulose reached 1.2%, the levels of expression of *FfCEL6B* and *FfMYB15* showed a downward trend, which were 1.69 and 1.68 times that in the control mycelium, respectively (Fig. 5a).

The levels of expression of *FfCEL6B* and *FfMYB15* in mycelia cultured on the 0.9% cellulose medium for 4, 5, 6, 7, and 8 d were detected. It was found that levels of expression of *FfCEL6B* and *FfMYB15* increased gradually during mycelial growth (Fig. 5b). These results indicated that the expression patterns of *FfCEL6B* and *FfMYB15* in mycelia cultured on the medium with different concentrations of cellulose for 8 d were similar with a correlation coefficient of 0.822. The expression patterns of *FfCEL6B* and *FfMYB15* in mycelia cultured on the 0.9% cellulose medium for different times were similar with a correlation coefficient of 0.953. These results further indicated that there may be a certain relationship between *FfCEL6B* and FfMYB15.

### Subcellular localization and analysis of transcriptional activity of FfMYB15

**Fig. 6:**
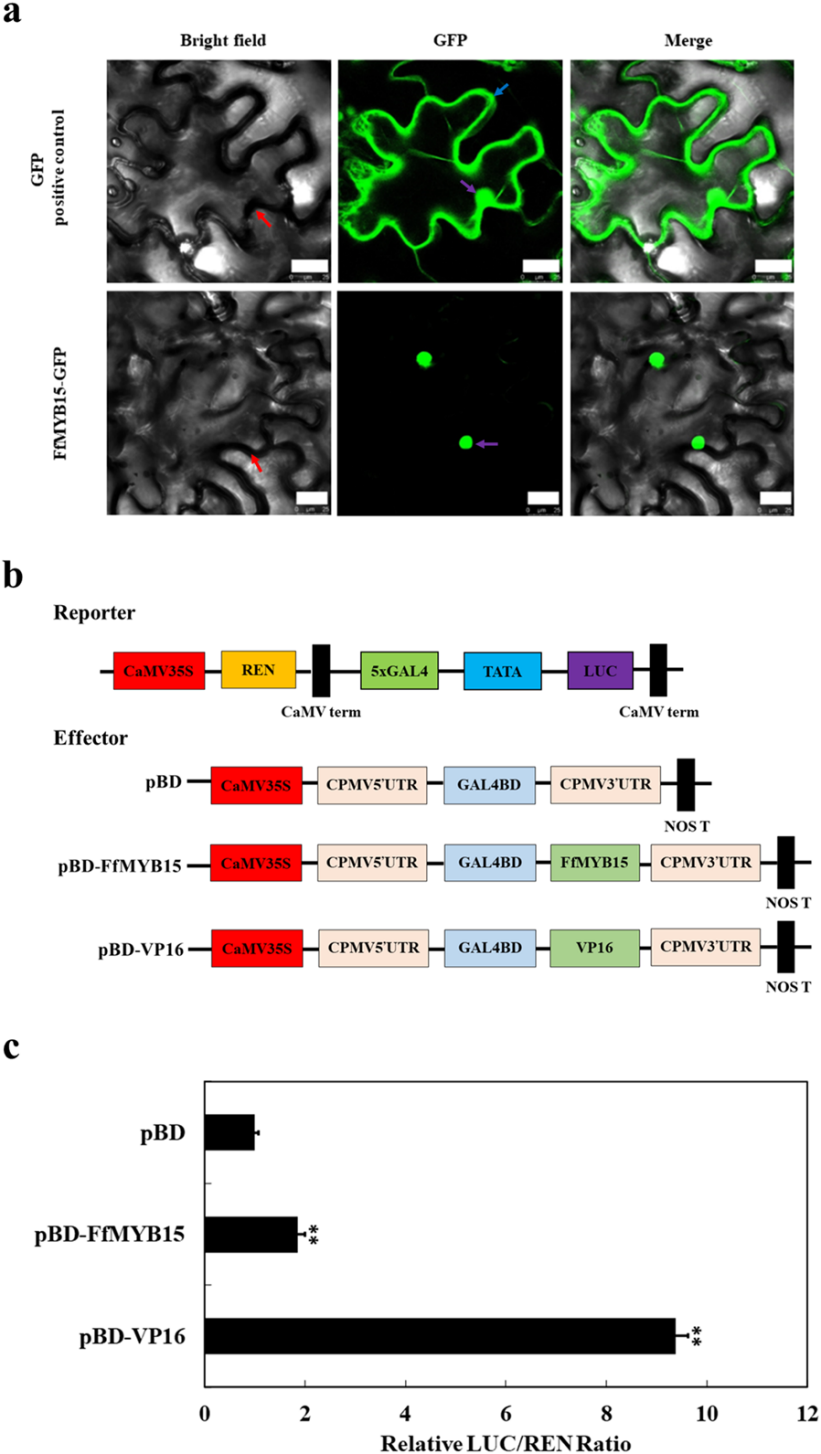
Subcellular localization and analysis of transcriptional activity of FfMYB15. **a** Subcellular localization. GFP (positive control) and FfMYB15-GFP in tobacco leaves 60 h after infiltration. Bar = 25 μm. The red arrow indicates the borders of cells. The blue arrow denotes the plasma membranes. The purple arrow denotes the nucleus. **b** Structural diagram of the effector and the reporter used in dual-luciferase assays. **c** Analysis of transcriptional activity of FfMYB15. The ratio of Luc/Ren of the empty pBD plasmid was used as standard and set to 1. Each value represents the mean ± S.E.M. (n = 6). **denotes a significant difference between the sample and the control at *P* < 0.01

TFs need to be transferred into the nucleus to fulfill their functions after synthesis in the cytoplasm [41]. To study the subcellular localization of FfMYB15, the cloned *FfMYB15* sequence was inserted into the pEAQ-GFP plasmid of green fluorescent protein (GFP), which was transformed EHA105 and was expressed instantaneously in tobacco leaves. After 60 h, this was recorded using a confocal laser scanning microscope. The green fluorescence occurred all over the cytoplasm and nucleus in the positive control. The green fluorescence was distributed in the nucleus in FfMYB15-GFP (Fig. 6a).

To understand the transcriptional activity of FfMYB15, the dual-luciferase reporter system was used in tobacco leaves. The pGreenII0800-LUC plasmid was modified as a reporter. pBD (negative control), pBD-FfMYB15, and pBD-VP16 (positive control) were used as effectors (Fig. 6b). These plasmids were transformed EHA105 and were expressed instantaneously in tobacco leaves. The results indicated that the firefly luciferase (Luc)/Renilla luciferase (Ren) ratio of pBD-FfMYB15 was significantly higher than pBD, but lower than pBD-VP16 (Fig. 6c). These results indicated that FfMYB15 was present in the nucleus and was a transcriptional activator.

### FfMYB15 binds to the *FfCEL6B* promoter and activates the transcription of *FfCEL6B*

**Fig. 7:**
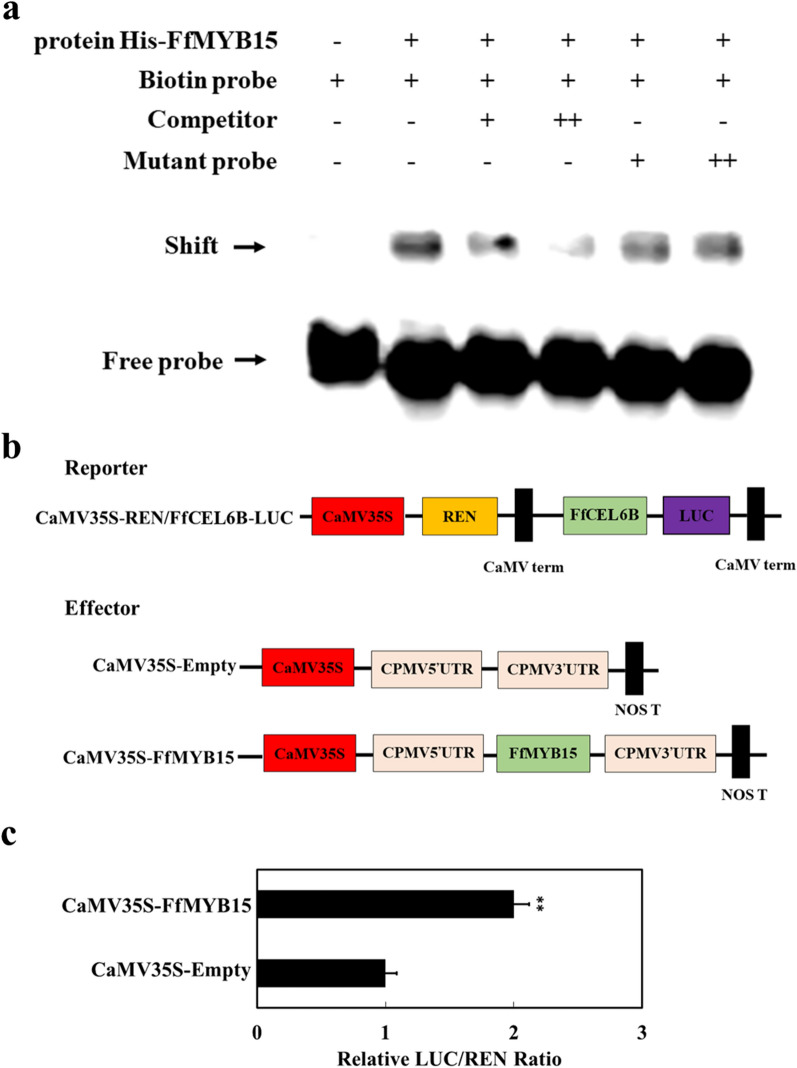
FfMYB15 could bind and activate *FfCEL6B* promoter. **a** EMSA. The purified FfMYB15 recombinant protein was used as a negative control alone. The symbols – indicate protein or probe not added. + and +  + represent increasing amounts of unlabeled or mutant probes used for competition and testing of binding. **b** Structural diagram of the effector and the reporter **c** FfMYB15 activates the transcription of *FfCEL6B*. The ratio of Luc/Ren of empty plasmid plus promoter was used as the standard and set to 1. Each value represents the mean ± S.E.M. (n = 6). **refers to statistically significant differences at *P* < 0.01

Only by combining MYB TFs with MYB motif of target gene promoter can the target gene be expressed and play a role [42, 43]. By using electrophoretic mobility shift assay (EMSA) to verify the binding of FfMYB15 to the *FfCEL6B*, it was found that only one probe band was displayed, when a biotin-labeled probe was added. When the FfMYB15 recombinant protein was incubated with this biotin-labeled probe (containing the MYB motif), the band-shift was found. When too many unlabeled probes (cold competitors) were added, the band-shift was effectively abolished. When many mutated probes (CAACCA was mutated to AAAAAA) were added, band shifts were not eliminated (Fig. 7a). These results indicated that FfMYB15 can bind to *FfCEL6B* promoter.

FfMYB15 had transcriptional activation activity and was bound to the *FfCEL6B* promoter. However, FfMYB15 activation of the transcription of *FfCEL6B* was unclear. Therefore, transient dual-luciferase assays were used to determine the ability of FfMYB15 to activate the transcription of *FfCEL6B*. The *FfCEL6B* promoter was inserted into pGreenII 0800-LUC plasmid as a reporter. The cloned *FfMYB15* sequence was inserted into the pEAQ plasmid. Recombinant plasmid and pEAQ plasmid (negative control) were used as effectors (Fig. 7b).

These plasmids were transformed EHA105 and were expressed instantaneously in tobacco leaves. Results showed that the transient expression of FfMYB15 significantly increased the Luc/Ren ratio of the reporter containing *FfCEL6B* relative to the negative control (Fig. 7c). These results indicated that FfMYB15 could bind and activate *FfCEL6B* promoter.

## Discussion

The growth rate and yield of mushrooms are directly related to the energy provided by cellulose degradation to glucose in the culture medium [44]. In this study, the growth rate of mycelia in the cellulose medium increased with gradually increasing cellulose concentration, and the growth rate reached a maximum at a concentration of 0.9%; however, with the continuous increase in concentration, the growth rate of mycelia showed a downward trend. The reason may be that the carbon nitrogen ratio was unbalanced with the excessive addition of cellulose, which affected the growth rate of mycelia.

The conversion of cellulose to glucose requires the combined action of a variety of cellulases [10]. Endoglucanase randomly hydrolyzes the β-1,4-glycosidic bonds inside the cellulose molecule, truncates the long-chain fiber molecule, and produces a large number of small molecules of cellulose with non-reducing ends. CBH acts on the non-reducing end of fiber molecules and hydrolyzes β-1,4-glycosidic bonds to form cellobiose molecules. BGL breaks down cellobiose to glucose [11]. Previous studies found that *CEL6B* gene encoding CBH existed in fungi (*Trichoderma viride* and *Thermobifida fusca*) and was involved in cellulose degradation [45, 46]. The *CEL6B* has also been identified in *L. edodes* and is involved in the degradation of cellulose [23]. In this study, we investigated *CEL6B* in *F. filiformis*. When the mycelia were cultured on the 0.9% cellulose medium, it was found that the levels of expression of *FfCEL6B* in mycelia gradually increased with prolonged culturation, which was consistent with the pattern of expression of *CEL6B* in *L. edodes* mycelia [18]. The analysis of RNAi showed that *FfCEL6B* could slow down the growth rate of mycelia, which indicated that *FfCEL6B* was involved in the degradation of cellulose and was positively correlated with the mycelial growth of *F. filiformis*. In the RNA-Seq study of *L. edodes*, it was found that the expression of cellulase gene was regulated by transcription factors, but the transcriptional regulation mechanism was unclear [18, 23], therefore, understanding the transcriptional regulation mechanism of cellulose degradation can provide a new basis for the growth mechanism of mushrooms.

MYB TFs play an important role in the growth and development of plants and filamentous fungi [47–49]. For example, cotton GhMYB7 participated in the regulation of secondary cell wall biosynthesis of cotton fiber through transcriptional activation of *AtSND1* and *AtCesA4* [47]. *Arabidopsis thaliana* MYB46 participated in the synthesis of cellulose in the secondary cell wall by transcriptionally activating three cellulose synthase (*CESA4*, *CESA7*, and *CESA8*) [48]. *Acremonium chrysogenum* AcMybA negatively regulated the transcription of cephalosporin biosynthetic genes *pcbC* and *cefEF*, thereby inhibiting the production of cephalosporin [49]. Similarly, recent studies found that MYB TFs also played an important role in mushroom growth and development. It has been reported that the MYB TFs of *P. ostreatus* and *G. lucidum* were involved in growth and development [27, 31]. Our study implied that there were also MYB TFs in *F. filiformis*. Among them, FfMYB15 was located in the nucleus, which was consistent with the result that 56 *MYB* genes in *G. lucidum* were located in the nucleus [27]. PoMYB TFs were found to participate in the growth and development of in *P. ostreatus* through the heat-stress pathway [31]. There was a cis acting element (CAT box) related to the growth and development process in the promoter region of *G. lucidum MYB* gene. The co-expression analysis of *G. lucidum MYB* gene showed that *MYB* was involved in the growth and development pathway of *G. lucidum* [27]. Our study differed from that; through transcriptional activity analysis, EMSA, and dual-luciferase transient expression assay, it was found that FfMYB15 can regulate the expression of *FfCEL6B* gene, participate in the cellulose degradation pathway, and then participate in the growth of *F. filiformis*.

MYB TFs regulated the expression of downstream target genes by binding to MYB motif in the promoter region of downstream target genes. For example, in persimmon fruit, DkMYB4 participated in proanthocyanidin biosynthesis by binding to MYB motif in the promoter region of proanthocyanidin pathway genes [50]. In the early stage of rice pollen development, GAMYB regulated the expression of bHLH142 by binding to the MYB motif in the promoter region of bHLH142 [51]. In *D. discoideum*, the MYB motif in the promoter of cellulase *CelB* might bind to MYB protein to regulate the *CelB* gene expression [30]. In this study, the regulation of *FfCEL6B* expression by FfMYB15 was also achieved through the binding of FfMYB15 to the MYB motif in the *FfCEL6B* promoter region.

In previous studies, cellulase genes were regulated by different transcription factors. For example, in *T. reesei*, Xyr1 and ACEI can regulate the expression of the cellulase *cbh1* gene, and ACE4 can regulate the expression of the cellulase gene by binding to the promoter of cellulase gene [52, 53]. In this study, we also found that FfMYB15 could regulate the expression of cellulase gene, which indicated that MYB can also participate in the cellulose degradation mechanism, however, the mechanism of cellulose degradation has not been thoroughly studied. Whether the RNAi and over-expression of *FfMYB15* will affect the expression of other cellulase genes remains unclear, therefore, the RNAi and overexpression transformants of *FfMYB15* should be obtained to explore the underlying regulatory mechanism.

## Conclusion

In this study, it was found that the *FfCEL6B* was actively involved in the mycelial growth of *F. filiformis*. In addition, a transcriptional activator, FfMYB15, was identified, which was localized to the nucleus and regulated *FfCEL6B* expression by binding to the promoter region of *FfCEL6B*. The findings reported here provided new information on the transcriptional regulatory mechanism of cellulose degradation during the growth of *F. filiformis*.

## Methods

### Strains and media

The strain F0027 of *F. filiformis* was provided by Shanxi Edible Fungi Germplasm Resource Collection Center of China. Mycelia of F0027 were cultured on the 0, 0.3%, 0.6%, 0.9%, and 1.2% cellulose medium (*w/v*, 0.15% peptone, 0.1% magnesium sulfate, 2% agar, and 0.05% potassium dihydrogen phosphate) respectively at 25 °C for 8 d in the dark.

*Escherichia coli* DH5α (TransGen Biotech, Beijing, China) and *Agrobacterium tumefaciens* EHA105 (Shanghai Weidi Biotechnology Co, Shanghai, China) were used for propagation of plasmids and transferring the plasmids into tobacco specimens, respectively.

### Detection of cellulase activity

The content of reducing sugar produced by cellulose degradation catalyzed by cellulase was determined by a 3,5-dinitrosalicylic acid method [36]. Enzyme activities were measured according to the instructions of the cellulase activity detection kit (Solarbio, Beijing, China). Fresh mycelium samples were weighed to about 0.1 g respectively, to which we added 1 ml extracting solution before homogenization in an ice bath. Reaction samples were centrifuged at 4 °C for 10 min, and the supernatant was taken and placed in an ice bath for testing. Reaction reagents were added to convert reducing sugar into 3-amino-5-nitrosalicylate. Absorbance values were detected at the wavelength of 540 nm using a spectrophotometer (Lab Tech, Beijing, China) [54]. The standard curve was plotted with 1 mg/mL anhydrous glucose as the standard. The regression equation was *y* = 17.683*x*–0.143, *R*^*2*^ = 0.9992. One enzyme activity unit was defined as the mass (g) of tissue catalyzed to produce 1 μg glucose per minute in the reaction system.

### Gene cloning and sequence analysis

Total RNA of mycelia was extracted using OMEGA E.Z.N.A. Plant RNA Kit (Omega, Bio-Tek, USA). RNA concentration and quality were assessed respectively by spectrophotometry and gel electrophoresis. cDNA was synthesized using HiScript II 1st Strand cDNA Synthesis Kit (Vazyme, Nanjing, China). The cDNA samples were diluted 10 times for gene cloning.

A cellulase gene and a MYB transcription factor were obtained from the genome of *F. filiformis* [35]. As this cellulase gene showed high degree of sequence homology to *Flammulina velutipes* CEL6B (E-value: 0.0; Per. Identity: 95.50%; GenBank: ADX07335.1), so it was named *FfCEL6B*. As this MYB transcription factor had high degree of sequence homology to *P. ostreatus* MYB15 (E-value: 0.0; Per. Identity: 72.16%; GenBank: axb87533.1), it was named FfMYB15. *FfCEL6B* and *FfMYB15* were cloned by gene-specific primer pairs and sequenced (Tsingke Biotechnology Co., Ltd, Beijing, China). Molecular weight, theoretical isoelectric point (pI), and nuclear localization sequence (NLS) were predicted by using the online NovoPro (https://www.novopro.cn/). DNA domains were predicted using conserved domain tool in NCBI (https://www.ncbi.nlm.nih.gov/Structure/cdd/wrpsb.cgi) and SMART (http://smart.embl.de/). Furthermore, sequences were aligned using DNAMAN [55]. Phylogenetic trees were constructed using MEGA-X (Statistical method: neighbor-joining; test of phylogeny: bootstrap method; number of bootstrap replications: 1000).

### Construction of RNAi-*FfCEL6B* plasmid and fungal transformation

The cloned *RNAi-FfCEL6B-Sense* and *RNAi-FfCEL6B-Anti* sequences were inserted into the modified pCAMBIA1300 plasmid provided by Ludan Hou, which contains hyg phosphotransferase gene (*hyp*) [55]. The plasmid was digested with restriction enzymes *Spe*I and *Bgl*II for homologous recombination the *RNAi-FfCEL6B-Sense* sequence, and then the recombinant plasmid was digested with *Spe*I and *Psp*OMI for homologous recombination the *RNAi-FfCEL6B-Anti* sequence. The *RNAi-FfCEL6B-Sense* sequence and *RNAi-FfCEL6B-Anti* sequence formed a hairpin structure. RNAi‑*FfCEL6B* plasmid was introduced into *A. tumefaciens* GV3101. According to a previous method [55], RNAi‑*FfCEL6B* plasmid was transferred into *F. filiformis* by *A. tumefaciens* GV3101. The positive transformants were detected by the polymerase chain reaction (PCR) with primers Ffhyg-F and Ffhyg-R.

### Gene expression analysis

By using reverse transcription-quantitative PCR (RT–qPCR) with the ChamQ™ Universal SYBR^®^ qPCR Master Mix (Vazyme, Nanjing, China) according to the manufacturer’s instructions, levels of expression of genes were detected. The qPCR was conducted on a Bio-Rad CFX Connect TM Real-Time PCR System (Bio-Rad, Hercules, CA, USA). Reaction mixtures (20 μL volume) contained 0.4 μL 10 μM of each primer, 10 μL ChamQ Universal SYBR qPCR Master Mix (2 ×), 4 μL cDNA template, and 5.2 μL ddH_2_O. The qPCR procedure was described as follows: initially denatured at 95 °C for 30 s, followed by 40 cycles to 95 °C for 10 s and 60 °C for 30 s, followed by final extension at 72 °C for 30 s. Each cDNA sample was analyzed in triplicate, and the average threshold cycle was calculated. *β-actin* (GenBank Accession No.AFM83888) in *F. filiformis* was used as the reference gene [56]. Levels of expression of *FfCEL6B* and *FfMYB15* were calculated using the 2^−ΔΔCt^ method [57].

### Subcellular localization of FfMYB15

The cloned *FfMYB15* sequence was inserted into pEAQ-GFP plasmid (*Age*I) provided by Bing Deng [58]. The GFP plasmid (positive control) and recombinant pEAQ-FfMYB15-GFP plasmid were transferred into EHA105, then transferred to the infiltration buffer (150 mM acetosyringone, 10 mM MES monohydrate, 10 mM magnesium chloride and pH 5.6) to adjust the OD600 value to 0.5. Finally, the buffer infiltrated into uniformly sized, well-growing tobacco (*Nicotiana benthamiana*) leaves [58]. Tobaccos were cultured in the dark for 12 h in order to resume leaf growth, and transferred to normal culture conditions. Transient expressions of GFP and pEAQ-FfMYB15-GFP were recorded by using a confocal laser scanning microscope (Leica, Weztlar, Germany) after 60 h.

### Transcriptional activity

The cloned *FfMYB15* sequence was inserted into the pBD plasmid (*Stu*I) provided by Bing Deng [58]. The recombinant pBD-FfMYB15, pBD (negative control), and pBD-VP16 (positive control) plasmid were used as effectors. The modified pGreenII0800-LUC plasmid was used as reporter, which contains Ren and Luc. Ren was driven by CaMV35S promoter and Luc was driven by the minimal TATA box of CaMV35S promoter with 5 × GAL4 binding elements. Reporters and effectors were transferred into EHA105 and transferred to the infiltration buffer to adjust the OD600 to 0.8. Effectors and reporters were mixed in the ratio of 9:1 and injected into uniformly sized, well-growing tobacco leaves [58]. Tobaccos were cultured in the dark for 12 h in order to resume leaf growth, and then transferred to an environment representing normal culture conditions. After 48–72 h, the ratio of Luc/Ren in tobacco leaves was detected by Double-Luciferase Reporter Assay Kit (TransGen Biotech, Beijing, China). Six independent replicates were assessed.

### *FfCEL6B* promoter analysis

The genomic DNA of *F. filiformis* was extracted by using Plant Genomic DNA Kit (TIANGEN, Beijing, China). The *FfCEL6B* promoter (1000 bp upstream of the initiation codon ATG) was cloned by gene-specific primer pairs and sequenced. Promoter elements of *FfCEL6B* were studied using the on-line Plant-CARE tool (http://bioinformatics.psb.ugent.be/webtools/plantcare/html/).

### Electrophoretic mobility shift assay (EMSA)

The cloned *FfMYB15* sequence was inserted into pCold I plasmid (*Kpn*I and *Pst*I) provided by Bing Deng and introduced into *Escherichia coli* BL21 (DE3) [58]. The His-FfMYB15 recombinant protein was expressed by 0.5 mM isopropyl β-D-thiogalactoside (IPTG) at 16 °C for 12 h. The His-FfMYB15 recombinant protein was purified by Ni–NTA Resin (TransGen Biotech, Beijing, China). The *FfCEL6B* promoter with 56 bp containing a MYB motif was end labeled with a biotin 3′ end DNA labeling kit (Beyotime, Shanghai, China). The biotin-labeled and unlabeled probes were incubated with purified FfMYB15 recombinant protein respectively for electrophoretic EMSA reaction. Results were detected by using a Light Shift Chemiluminescent EMSA kit (Beyotime, Shanghai, China).

### Dual-luciferase reporter assay

The *FfCEL6B* promoter (1000 bp) was inserted into the pGreenII0800-LUC plasmid as a reporter. The cloned *FfMYB15* sequence was inserted into the pEAQ plasmid as an effector. All the above plasmids were transferred into EHA105 and transferred to the infiltration buffer to adjust the OD600 to 0.8. The effector and the reporter were mixed in the ratio of 9:1 before injection into tobacco leaves, which were assayed by Double-Luciferase Reporter Assay Kit (TransGen Biotech, Beijing, China) after 48–72 h. Six independent replicates were assessed.

### Statistical analysis

All analyzed data were biologically repeated at least three times. Data represent the mean ± S.E.M or mean ± SD. Significant differences were determined using SPSS software. Analysis of variance (ANOVA) and Duncan’s multiple-range tests were performed to evaluate differences between the samples. The Pearson correlation coefficient method (*P* < 0.05) was used for calculating the correlation coefficient.

## Supplementary Information

**Additional file 1: Table S1.** Primers used in this study. **Figure S1.** Cellulase activity of mycelia cultured on the medium with 0, 0.3%, 0.6%, 0.9%, and 1.2% concentrations of cellulose for 3, 4, 5, 6, 7, and 8 days. Each value represents the mean ± SD (n = 3), and different lowercase letters denote statistical significance (*P* < 0.05). **Figure S2.** Cloning of *FfCEL6B*. **Figure S3.** Cloning of *FfMYB15*.

## Data Availability

All the research results and data in this work have been included in the manuscript and supplementary materials. The original data involved in this study can be obtained from the first author or corresponding author through email upon reasonable request.
