# Supplementary material for: Transcription factor FfMYB15 regulates the expression of cellulase gene FfCEL6B during mycelial growth of Flammulina filiformis
Source: Microb Cell Fact. 2022 Oct 17;21:216. doi: 10.1186/s12934-022-01932-z (PMC9578197; doi:10.1186/s12934-022-01932-z)
Supplement: Supplementary file 1 — Additional file 1: Table S1. Primers used in this study. Figure S1. Cellulase activity of mycelia cultured on the medium with 0, 0.3%, 0.6%, 0.9%, and 1.2% concentrations of cellulose for 3, 4, 5, 6, 7, and 8 days. Each value represents the mean ± SD (n = 3), and different lowercase letters denote statistical significance (P < 0.05). Figure S2. Cloning of FfCEL6B. Figure S3. Cloning of FfMYB15. [file 12934_2022_1932_MOESM1_ESM.docx]

**Additional file**

**Table S1** Primers used in this study.

| Primer | Sequence (5′→3′) | Note |
| --- | --- | --- |
| *FfCLE6B*-F | ATGTTGAACTCTCTGGCTCT | cDNA fragment cloning |
| *FfCLE6B*-R | TTACAAGGGAGGGTTAGCCT |  |
| *FfMYB15*-F | ATGGCTATCTTTCAAGAGGACTA |  |
| *FfMYB15*-R | TTAATCTTCAGCAAAATTGGCGTT |  |
| pCold I*-FfMYB15*-F | ATCGTGACGAGCTCGGTACCATGGCTATCTTTCAA | Construction of pCold I plasmid |
| pCold I*-FfMYB15*-R | ATTACCTATCTAGACTGCAGTTAATCTTCAGCAAA |  |
| pEAQ-GFP*-FfMYB15*-F | TGCCCAAATTCGCGACCGGTATGGCTATCTTTCAA | Construction of pEAQ-GFP plasmid |
| pEAQ-GFP*-FfMYB15*-R | CCTTTGCTAGTCATACCGGTATCTTCAGCAAAATT |  |
| pBD*-FfMYB15*-F | TATCGCCGACCGGTAGGCCTATGGCTATCTTTCAA | Construction of pBD plasmid |
| pBD*-FfMYB15*-R | GAAACCAGAGTTAAAGGCCTATCTTCAGCAAAATT |  |
| pEAQ*-FfMYB15*-F | TGCCCAAATTCGCGACCGGTATGGCTATCTTTCAA | Construction of pEAQ plasmid |
| pEAQ*-FfMYB15*-R | CCAGAGTTAAAGGCCTCGAGATCTTCAGCAAAATT |  |
| RNAi-*FfCEL6B*-Sense-F | CTATTATAAGACTAGTAGGAACCTATCTCGC | Construction of RNAi plasmid |
| RNAi-*FfCEL6B*-Sense-R | CGATGATTGTAGATCTGTTACTGGGTCGGGC |  |
| RNAi-*FfCEL6B*-Anti-F | CTATTATAAGACTAGTGTAGCCAATCCACGGAAGTAAGGT |  |
| RNAi-*FfCEL6B*-Anti-R | CAATTCTAGAGGGCCCAGGAACCTATCTCGCTGATGCTTC |  |
| Ffhyg-F | CGACAGATCCGGTCGGCATCTACTCTATTTCTT | Detection of transformants |
| Ffhyg-R | TCTCGTGCTTTCAGCTTCGATGTAGGAGGG |  |
| *FfCLE6B*-qdF | TTCGACTTTAGTACGTGGCGTC | gDNA fragment cloning |
| *FfCLE6B*-qdR | GTTGGAAATGCTGTATCCTTGTCC |  |
| pGreenII0800-LUC*-FfCLE6B*-F | TATAGGGCGAATTGGTTCGACTTTAGTACG | Construction of pGreenII0800-LUC plasmid |
| pGreenII0800-LUC*-FfCLE6B*-R | TTGGCGTCTTCCATGGCATGTTGGAAATGCT |  |
| *FfCLE6B-*Probe-F | TTGCCACCTCAACTCCTCTAGCTCCCAACCATCAGTTTCCTATCTCAAGGAAGTTT | Probe of promoter |
| *FfCLE6B-*Probe-R | AAACTTCCTTGAGATAGGAAACTGATGGTTGGGAGCTAGAGGAGTTGAGGTGGCAA |  |
| *FfCLE6B-*Mutant-F | TTGCCACCTCAACTCCTCTAGCTCCAAAAAATCAGTTTCCTATCTCAAGGAAGTTT |  |
| *FfCLE6B-*Mutant-R | AAACTTCCTTGAGATAGGAAACTGATTTTTTGGAGCTAGAGGAGTTGAGGTGGCAA |  |
| *β-actin*-F | TACCCATACCGTTCCCATCT | Endogenous control |
| *β-actin*-R | ACCCACGCTCCATGAGGT |  |
| *FfCLE6B*-qF | ACACGAAGCATCATCAGACCTA | qPCR |
| *FfCLE6B*-qR | GAAGAGCGAGTGGTTGATATGG |  |
| *FfMYB15*-qF | GTTCTCCTAGCAGCGAAGA |  |
| *FfMYB15*-qR | CATTCCCCGTTTTAAGTGAC |  |


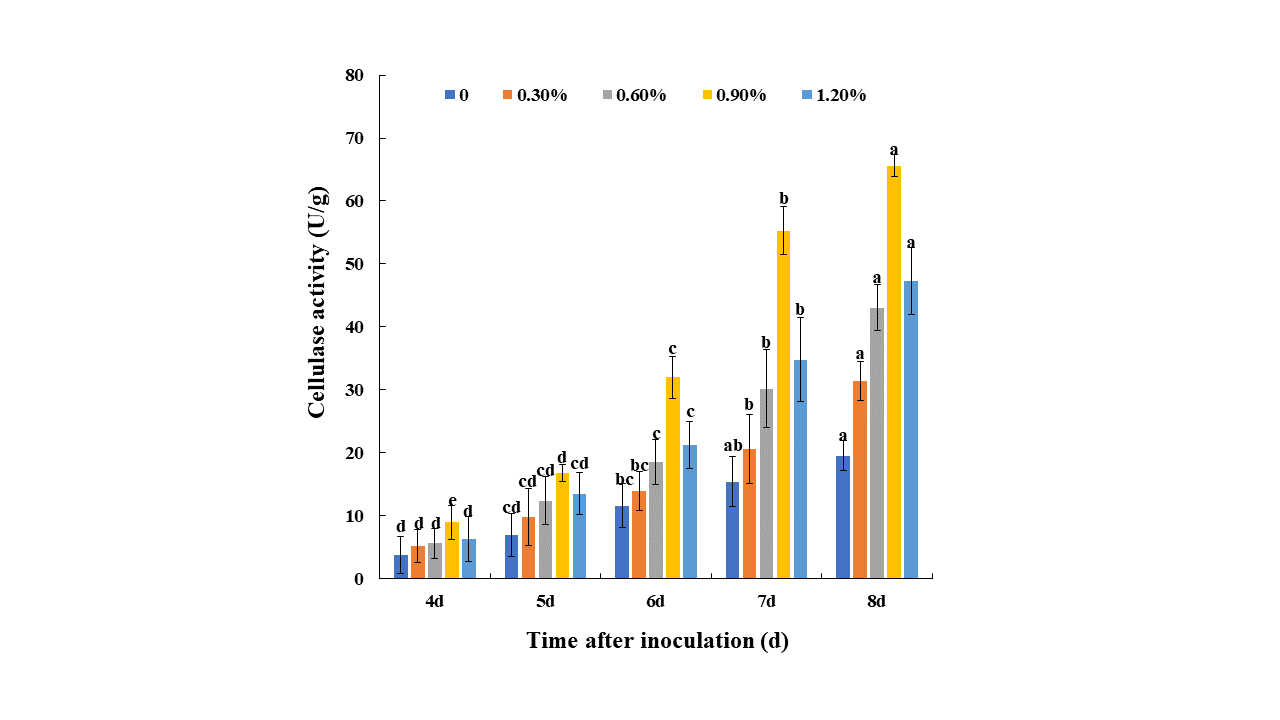


Figure S1. Cellulase activity of mycelia cultured on the medium with 0, 0.3%, 0.6%, 0.9%, and 1.2% concentrations of cellulose for 3, 4, 5, 6, 7, and 8 days. Each value represents the mean ± SD (n = 3), and different lowercase letters denote statistical significance (*P* < 0.05).


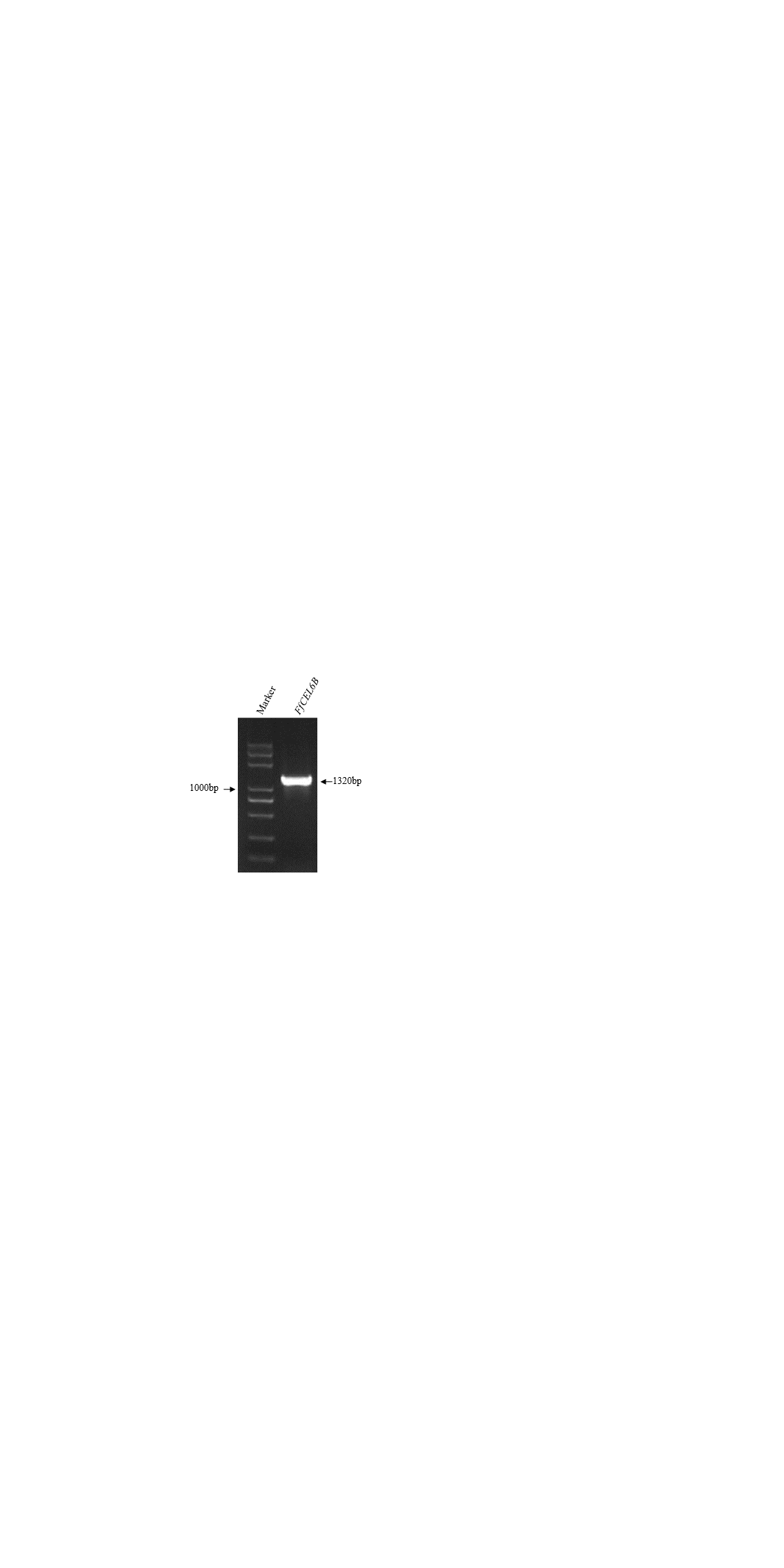

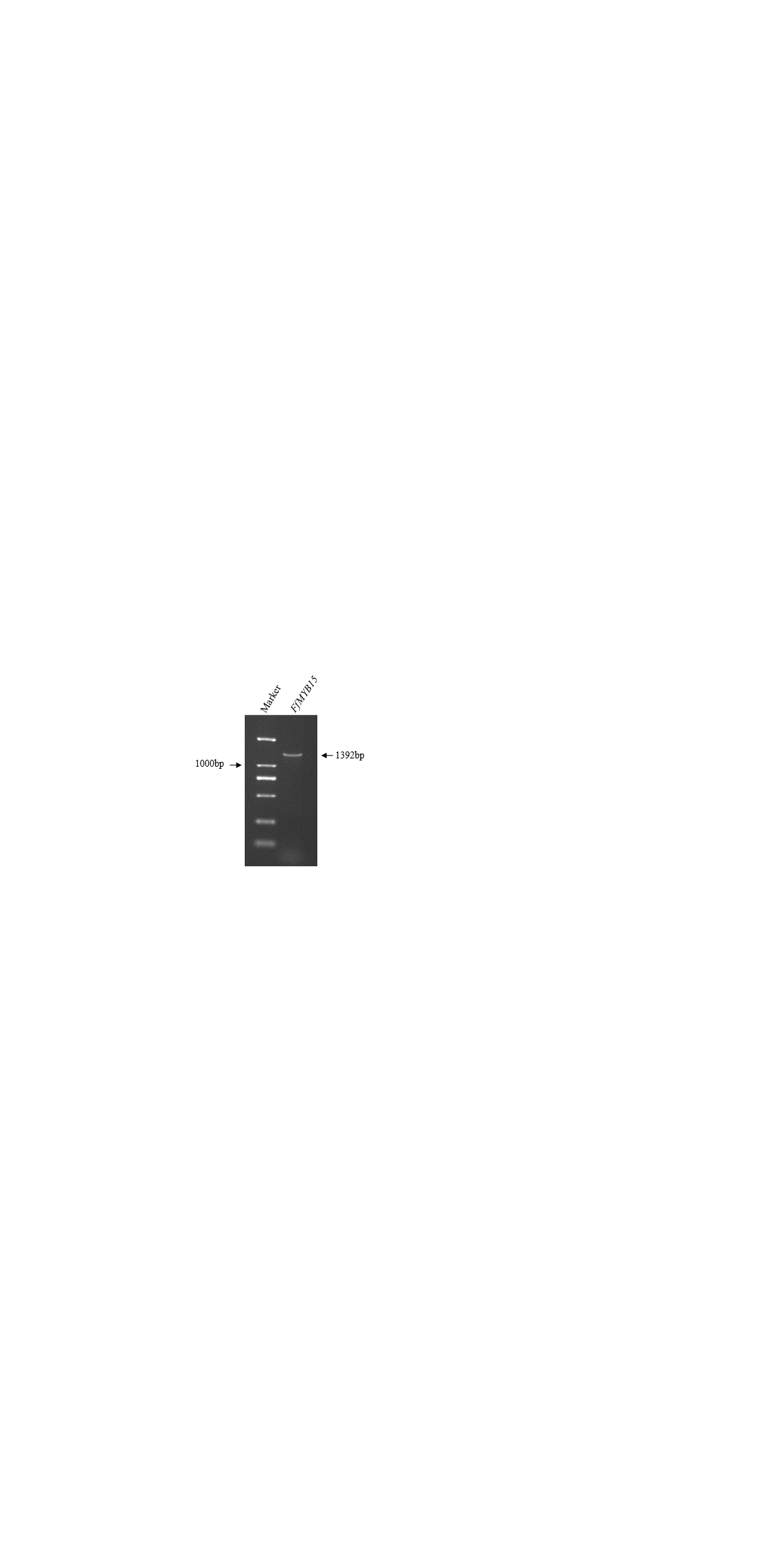


Figure S3. Cloning of *FfMYB15*

Figure S2. Cloning of *FfCEL6B*
